# Establishing the efficacy of interventions to improve health literacy and health behaviours: a systematic review

**DOI:** 10.1186/s12889-020-08991-0

**Published:** 2020-06-30

**Authors:** Ronie Walters, Stephen J. Leslie, Rob Polson, Tara Cusack, Trish Gorely

**Affiliations:** 1 Centre for Health Science, University of the Highlands and Islands, Old Perth Road, Inverness, IV2 3JH UK; 2 NHS Highlands, Cardiology Department, Raigmore Hospital, Old Perth Road, Inverness, IV2 3JH UK; 3 University College Dublin, Health Sciences Building, Bellfield, Dublin, Ireland

**Keywords:** Health literacy, Behaviour change, Cardiovascular disease, Systematic review, Interventions

## Abstract

**Background:**

The primary aim of this review was to establish whether health literacy interventions, in adults, are effective for improving health literacy. Two secondary aims assessed the impact of health literacy interventions on health behaviours and whether health literacy interventions have been conducted in cardiovascular patients.

**Methods:**

A systematic review (Prospero registration: CRD42018110772) with no start date running through until April 2020. Eligible studies were conducted in adults and included a pre/post measure of health literacy. Medline, Embase, Eric, PsychINFO, CINAHL, Psychology and Behavioural Science, HMIC, Web of Science, Scopus, Social Care Online, NHS Scotland Journals, Social Policy and Practice, and Global Health were searched. Two thousand one hundred twenty-seven papers were assessed, and 57 full text papers screened to give 22 unique datasets from 23 papers. Risk of bias was assessed regarding randomisation, allocation sequence concealment, blinding, incomplete outcome data, selective outcome reporting and other biases. Intervention reporting quality was assessed using the TIDieR checklist.

**Results:**

Twenty-two studies were included reporting on 10,997 participants in nine countries. The majority of studies (14/22) were published in 2018 or later. Eight studies (n = 1268 participants) also reported on behavioural outcomes. Health literacy interventions resulted in improvements in at least some aspect of health literacy in 15/22 studies (n = 10,180 participants) and improved behavioural outcomes in 7/8 studies (n = 1209 participants). Only two studies were conducted with cardiovascular patients. All studies were at risk of bias with 18 judged as high risk. In addition, there was poor reporting of intervention content with little explication of the theoretical basis for the interventions.

**Conclusions:**

Health literacy interventions can improve health literacy and can also lead to changes in health behaviours. Health literacy interventions offer a way to improve outcomes for populations most at risk of health inequalities. Health literacy is a developing field with very few interventions using clear theoretical frameworks. Closer links between health literacy and behaviour change theories and frameworks could result in higher quality and more effective interventions.

**Prospero registration:**

Prospero registration: CRD42018110772

## Background

Health literacy as a concept first emerged in the 1970’s [1] and the definition further refined in 2000 by Nutbeam [2] who added interactive health literacy and critical health literacy to the existing focus on functional health literacy. These three aspects have endured throughout the subsequent developments of health literacy and are conceptualised as representing different levels of skills and understanding that move progressively towards greater autonomy and empowerment. They start from a base of functional health literacy (basic ability to read and understand health information [2, 3]) through interactive health literacy (more advanced cognitive and social skills that demonstrate greater engagement with a wider variety of health information, improved self-efficacy, and decision making [2–6]) and finally critical health literacy (higher order cognitive and critical decision making skills, alongside social, political and organisational level actions to improve wider determinants of health [2–7]).

In 2012 the European Health Literacy Consortium [6] conducted a major review of the literature and developed a new definition of health literacy - *“Health literacy is linked to literacy and entails people’s knowledge, motivation and competences to access, understand, appraise, and apply health information in order to make judgments and take decisions in everyday life concerning healthcare, disease prevention and health promotion to maintain or improve quality of life during the life course.” (Pg.3).* Applying this definition suggests that health literacy pervades patient’s encounters with healthcare services at every level. For a while the focus was on health literacy as a skill or asset that the patient was required to improve [8]. More recently there has been a recognition of the responsibility that healthcare services have to ensure they are providing information in a way that patients can understand [9].

### Health literacy and cardiovascular disease

The European health survey found that almost half of adults in eight countries had inadequate or problematic health literacy [10]. Weak health literacy competencies can result in increased rates of hospital readmission, low health related quality of life (HRQOL), higher anxiety levels and lower social support [11], less healthy choices, and poorer self-reported health status [12]. Health literacy is also a key predictor of self-assessed health second only to age [13].

Other studies have shown that patients with chronic conditions have lower levels of health literacy, and amongst this sector, cardiovascular patients have the highest number of problems understanding health information [14, 15]. Given this limitation, it is possible that interventions to improve health literacy may be important to support and facilitate subsequent behaviour change.

Cardiovascular diseases are the leading cause of mortality worldwide, responsible for 31% of deaths in 2015 [16]. Cardiovascular diseases are predominantly the result of lifestyle behavioural factors such as poor diet, inadequate physical activity, smoking or harmful alcohol consumption. Other physiological factors include high blood pressure, high cholesterol, high blood sugar or glucose. Both the physiological and the behavioural factors are linked with socio-economic and societal drivers such as ageing, income, location, education and housing [17].

Following a cardiac diagnosis there is a need for patients to learn to self-manage their condition, and for many a change in lifestyle could reduce the risk of further cardiovascular events. This can be supported through a course of cardiac rehabilitation offered through the national health service (NHS), though uptake is low [18]. Whilst there are many reasons why this may be the case [19–22], some of it may be due to inadequate levels of health literacy.

There has been a significant increase in the amount of research conducted into health literacy in recent years, including a much higher proportion with a European focus, however, much of this has been focused on identifying definitions, prevalence and associations [23]. Less research has been conducted into possible interventions for health literacy – in any health condition, and particularly within cardiac populations [24].

### Limitations with previous reviews

From a pool of 96 reviews in the field of health literacy none focused specifically on health literacy interventions in cardiac patients. Four reviews focused on health literacy in cardiac populations [25–28] and covered aspects such as prevalence, adherence to medication and measurement tools. Whilst some of these did include interventions, none required pre-post measures as an inclusion criterion. To be certain that a specified intervention has made a change to the outcome of interest (in this case health literacy) measuring before and after the intervention is an essential requirement. Including a control condition, increases the likelihood that outcomes are a result of the intervention content, rather than the contact the intervention brought. Both of these requirements are missing in all reviews identified by the search team.

### Aim

Many chronic or long-term health conditions such as cardiovascular disease, benefit from behavioural changes to support lifestyle modification. Improving health literacy skills is believed to result in patients being better able to manage lifestyles, seek information and have the confidence to apply it. As a consequence, improved health literacy may result in improved behavioural outcomes such as smoking cessation, increased physical activity, improved diet quality, successful weight management and reduced alcohol consumption.

The primary aim of this review is to establish whether controlled health literacy interventions, in adults, are effective for improving health literacy. Two secondary aims, using the studies identified for the primary aim, are to explore whether 1) health literacy interventions lead to a change in health behaviours and 2) which of the eligible studies were conducted with cardiovascular patients and examine the outcomes in this population.

## Methods

The protocol was registered with PROSPERO (registration number: CRD42018110772).

### Eligibility criteria

#### Inclusions

Eligible papers included any full text articles published in peer reviewed journals with adults (aged 18 or over) as the subject of the intervention (as opposed to parents/caregivers). Searches were restricted to English language only due to the capacity of the review team to translate or work with other language texts. Eligible interventions included any intervention evaluated in a controlled trial that included a pre-post measure of health literacy. Eligible control conditions include any usual care or alternative approach to the intervention. Primary outcomes were self-reported or objectively measured health literacy measured at baseline and post-intervention (either directly after intervention completion or at follow up, regardless of the duration). Secondary outcomes of interest for this study are changes in behavioural outcomes such as health screening, smoking, nutrition, alcohol or physical activity behaviours. In common with Nutbeam et al. (2018) we excluded mental health literacy interventions as the field is conceptually distinct from health literacy.

#### Exclusions

Papers were excluded if they were not available in English to allow the review team to effectively review them, if they did not report full peer reviewed results of an intervention (for example abstracts, unpublished studies, protocols), or if they did not report both pre and post measures of health literacy. It is worth noting that many of the excluded papers were either observational/correlational studies, or they only used health literacy to segment the intervention population, or the intervention was designed to improve health literacy but they did not measure health literacy as an outcome of interest. None of these studies were eligible for this review.

### Information sources

Searches were conducted on electronic databases with no start date restriction through to 10th April 2020. The following databases were searched: Medline, Embase, Eric, PsychINFO, CINAHL, Psychology and Behavioural Science, HMIC, Web of Science, Scopus, Social Care Online, NHS Scotland Journals, Social Policy and Practice, Global Health. Full search strategies for each database can be found in additional file 1. In addition, the references of the papers included in the systematic review were searched, along with several published systematic reviews in related areas to make sure no relevant articles were missed.

### Search strategy

Initial scoping searches to develop the search strategy included cardiology specific terms. The search strategy was then broadened and the results backchecked to ensure the strategy was still retrieving the cardiology related materials. Searches included a combination of terms from MESH headings and keywords in the title and abstract. The search included multiple terms for health literacy (e.g. health literacy, functional health literacy), intervention (e.g. intervention, pre-post, trial) and health literacy measurements (e.g. health literacy screen, health literacy measurement, REALM, TOFHLA). All terms within each category were combined with “OR” and then the three categories were combined with “AND”. The search strategy was created by RW, TG and RP (an experienced information specialist) and run by RW. Detailed search strategies can be found in supplementary Table 1 (additional file 1).

### Study selection

Search results were imported into Endnote X9 reference management software and duplicates were removed. The remaining papers were exported to RAYYAN [29] (a systematic review web application) and titles and abstracts screened through application of the inclusion/exclusion criteria by RW, with a random 10% screened independently (TG) with 100% concordance. Full texts of potentially relevant studies were screened independently by two reviewers (RW, TG). All included texts had the references hand searched to check for additional eligible papers.

### Data extraction

All data from included studies were extracted into Word independently by two reviewers (RW, TG). Details extracted included study details, (design, population), health literacy details (definition, measure of health literacy used, aspects of health literacy measured), intervention details (intervention content, contact time, aspect of health literacy targeted in the intervention) and outcomes (health literacy and secondary behavioural outcomes).

### Data analysis

All papers were assessed using the template for intervention description and replication (TIDieR) independently by RW and TG with 84% initial concordance, rising to 100% following discussion. Quality appraisal was conducted independently using risk of bias in non-randomised studies of interventions (ROBINS-1) for non-randomised controlled trials and the appropriate version of risk of bias 2 (RoB 2.0) for individual randomised, cluster randomised and cross-over trials. Earlier versions were adjusted in domain 5 to provide consistency with the questions in the latest version for randomised controlled trial (RCT) studies. Initial inter-rater agreement was 91% for overall risk of bias and 83% for sub-domains. After discussion all differences were resolved with 100% agreement.

Results are presented using a narrative synthesis as the variation in definitions and measurements rendered a meta-analysis unsuitable.

## Results

### Study characteristics

**Fig. 1:**
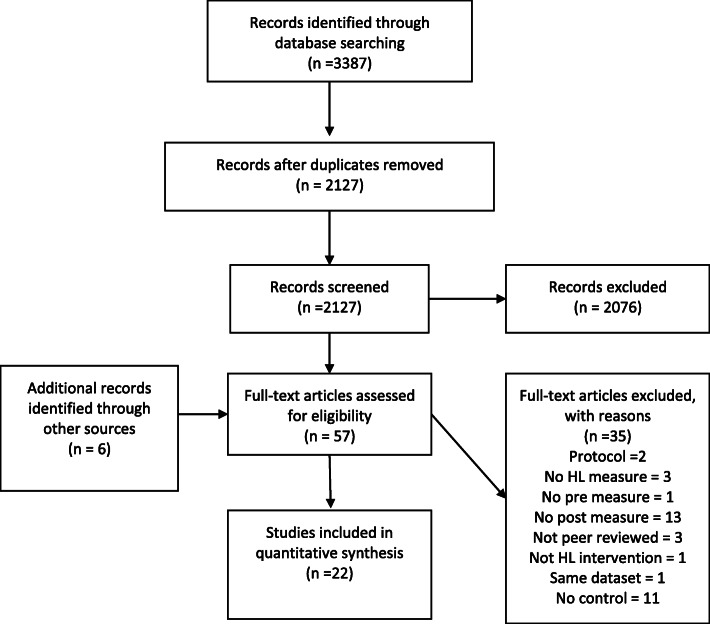
PRISMA Flow Diagram

This systematic review identified 3387 papers. After the removal of duplicates 2127 unique publications were screened and 2076 excluded based on title or abstract because they did not meet the inclusion criteria (see additional file 1 for detailed breakdown). Fifty-seven papers were retrieved for full text assessment, of which 35 failed to meet the inclusion criteria. Twenty-three papers [30–52] exploring 22 data sets (summarised in Table 1) were included in the final review (see Fig. 1). The two papers by Mas et al. [45, 46] were confirmed by the authors to relate to the same data set. Information from both papers was extracted to complete the review, however for clarity the reference for the latest paper will be used [45].

**Table 1:** Summary of studies

Reference, 1st author, year, country	Study design	Population: (Age, ethnic/migrant focus, n (f = %)	Measure [Objective/Subjective]	Health condition	Domain	Intervention content [Total in-person contact time^a^ (where applicable)]	Secondary Behavioural Outcomes measured
**Domain**	**Health Care**						
[30] Calderon, 2014 USA	RCT	Adults, Latino/Hispanic 240 (f = 81.66)	Diabetes Health Literacy Scale [Subjective]	Diabetes	Health care	13-min animation [n/a]	None
[31] Tai, 2016 USA	RCT	> 55 None 118 (f = 62.71)	STOFHLA [Objective]	Taking 2 or more medications	Health care	A single one-to-one 10-min education session plus leaflets and sample labels for self-assessment [10 min]	None
[32] Gharachourlo, 2018 Iran	RCT	18–35, None, 100 (f = 100)	Iranian Health Literacy Questionnaire [Subjective]	Gestational diabetes	Health care	Counselling with a health literacy approach (6 X 1.5 h weekly sessions) [540 min]	Health lifestyle behaviours
[33] Knudsen, 2019 Denmark	Quasi-experimental	Adults, None, 77 (f = 9)	HLQ [Subjective]	PCI, CABG, or left heart valve surgery	Health care	12 weeks of supervised exercise training, dietary advice, educational sessions and psychosocial support [unclear]	None
[34] Tavakoly Sany, 2019 Iran	RCT	30–75, None, 80 (f = 73.75%)	TOFHLA [Objective]	Heart failure	Health care	Three educational group workshops [200 min]	Self-care behaviours
[35] Banbury, 2020 Australia	Quasi-experimental	> 50, None 111 (f = 64.86)	HLQ [Subjective]	One or more chronic conditions	Health care	5 facilitative learning video-conference self-management group sessions [337.5 min]	None
[36] Handa, 2020 Japan	RCT	Adults, None 102 (f = 100)	HLS-14 [Subjective]	Breast cancer	Health care	Smartphone app for self-directed use to record symptoms and manage chemotherapy side-effects [n/a]	None
[37] Kim, 2020 USA	RCT	> 35, Korean American 209 (f = 40.9)	REALM, DM-REALM, TOFHLA, NVS [Objective]	Type 2 Diabetes	Health care	6 × 2 h weekly education sessions, monthly motivational interviewing counselling sessions and daily self-monitoring of blood glucose levels [1050 min^b^]	None
**Domain**	**Disease prevention**						
[38] Li, 2016 Niger	RCT	Working age adults, Chinese, 1441 (f = 2.98)	Malaria Health Literacy Questionnaire [Unclear]	Malaria	Disease prevention	Malaria prevention and treatment messages via WeChat 3xweek for 4 months [n/a]	None
[39] Han, 2017 USA	Cluster RCT	21–65, Korean American, 560 (f = 100)	Assessment of health literacy in cancer screening [Objective]	Cancer screening behaviours	Disease prevention	CHW provided education (Single 1.5–2-h small group session), monthly telephone counselling and navigation assistance over 6 months [155 min]	Cancer screening
**Domain**	**Health promotion**						
[40] Otilingam 2015, USA	RCT	> 40, Latino, 100 (f = 100)	NVS [Objective]	None	Health promotion (nutrition)	Two 2 h workshops (1 week apart) with culturally relevant nutrition education techniques and 3 behaviour change principles [240 min]	Nutrition
[41] Zhuang, 2016 China	Cluster RCT	Adults, None 6413 (f = unknown)	RAHL [Objective]	None	Health promotion (general)	Health education SMS message once a week for 1 year [n/a]	None
[42] Mas, 2017 USA	Quasi-experimental	21 or over, Hispanic, 97 (f = 81.44)	STOFHLA [Objective]	None	Health promotion (general)	ESL curriculum (180 h) with HL content (5 × 2 h sessions) [600 min^c^]	None
[43] Parekh, 2017 USA	Pilot RCT	Adults, None, 59 (f = 100)	NVS [Objective]	Cancer survivors	Health promotion (nutrition)	6 Small group nutrition education sessions (2 h duration) delivered fortnightly [720 min]	Nutrition
[44] Liu, 2018 China	Cluster RCT	60 and above, None, 260 (f = 49.23)	Chinese Citizen health literacy questionnaire [Unclear]	None	Health promotion (general)	Teach back educational classes 40 min long once a month for 6 months [240 min]	None
[45, 46] Mas, 2018 (and 2015) USA	RCT	Adults, Hispanic, 155 (f = 80.65)	TOFHLA [Objective]	None	Health promotion (general)	ESL curriculum with HL content. 12 units over 6 weeks for total of 36 h (6 h sessions) [2160 min]	Cardiovascular health behaviours
[47] Panahi, 2018 Iran	Cluster RCT	University Students, None, 130 (f = 60)	Health Literacy in Iranian Adults [Subjective]	Smoking prevention	Health promotion (smoking)	Six educational sessions via telegram Social media application [n/a]	Smoking
[48] Tsai, 2018 Taiwan	Quasi-experimental	Adults, Vietnamese/Indonesian 223 (f = 100)	Bespoke [Subjective]	None	Health promotion (general)	10 session (2 h per session) every other week, problem-based learning HL program [1200 min]	None
[49] Uemura, 2018 Japan	RCT	> 65, None 84 (f = 70.23)	HLS-EU-Q16 and HLS-14 [Subjective]	None	Health promotion (nutrition and physical activity)	Weekly 90-min active learning program for 24 weeks [2160 min]	Nutrition and physical activity
[50] Fiedler, 2019 Germany	Crossover RCT	Adults, None 72 (f = 19.44)	German Health Literacy Questionnaire [Subjective]	None	Health promotion (general)	5-month training program. Three modules delivered over 3-day classroom training, peer coaching and practice material. [1260 min]	None
[51] McCaffery, 2019 Australia	Cluster RCT	> 16, None, 213 (f = 70)	HLQ [Subjective]	None	Health promotion (general)	18 week health literacy focused adult education curriculum [6480 min]	None
[52] Smith, 2019 Australia	RCT	> 55, None, 153 (f = 66.66)	HLQ [Subjective]	None	Health promotion (general)	DVD/web based multi-media education on complementary medicine [n/a]	None

^a^In-person contact time is calculated through description provided in the original paper. Number of sessions x duration of sessions gives total in-person contact time over the duration of the intervention. If a range is given (e.g. 1.5–2 h) then the midpoint is taken

^b^This reflects 720 min of group education sessions and an average of 30 min per counselling session for 11 months

^c^This reflects active health literacy content, but was embedded within a wider ESL intervention of 10,800 min

Of the 22 included studies, the earliest study was from 2014 [30], the latest from 2020 [35–37] with the majority (fourteen) being published since 2018 [32–37, 44, 45, 47–52]. Eighteen studies were randomised and included 1 cross over design [50], 5 cluster randomised [39, 41, 44, 47, 51] and 12 individually randomised trials [30–32, 34, 36–38, 40, 43, 45, 49, 52]. The remaining four were quasi-experimental pre-post controlled trials [33, 35, 42, 48]. Studies took place in nine countries –USA [30, 31, 37, 39, 40, 42, 43, 45], Australia [35, 51, 52], Iran [32, 34, 47], China [41, 44], Japan [36, 49], Taiwan [48], Niger [38], Germany [50] and Demark [33].

Two studies focused on the domain of disease prevention (cancer screening [39] and malaria health literacy [38]), eight focused on healthcare (diabetes [30, 37], gestational diabetes [32], breast cancer [36], cardiac conditions [33, 34], one or more chronic conditions [35] and people taking two or more medications daily [31]). The remaining 12 studies focused on health promotion with eight focusing on general health promotion [41, 42, 44, 45, 48, 50–52], two focusing on nutrition [40, 43], one on nutrition and physical activity [49] and one on smoking prevention [47]. Studies are summarised in Table 1.

#### TIDieR assessment

Reporting was adequate for 58% of the intervention conditions and 57% of the control conditions. Six studies planned to tailor the intervention but only two reported adequately. Only one study modified the delivery of the intervention.

Across the 22 studies only seven reported on planned fidelity checks (reporting was adequate for four of the studies and unclear for the other three). Of these seven only two reported on actual fidelity, with only one being sufficiently clear to be considered adequate. Studies were generally good at providing a descriptive name or phrase (95%), rationale (100%), and details of how (86% for intervention, 69% for control) and frequency and intensity (73% for intervention, 64% for control). Planned fidelity (18%) and actual fidelity (5%) were the most poorly described. It should also be noted that whilst materials were described in 95% of intervention conditions and 69% of control conditions the percentage that were adequately reported was much lower (41% of intervention, 38% of control). See supplementary Tables 2 & 3 (additional files 2 & 3) for TIDieR reporting and percentage summary tables.

#### Risk of Bias

No studies were rated at a low risk of bias overall, with 18 being at a high or serious risk of bias and just four being rated at some concerns (see supplementary Table 4 (additional file 4)). Studies were generally poorly rated in the randomisation process with only three being at low risk of bias. The majority of studies showed low risk of bias for the deviations from intended intervention (20 studies). Thirteen studies had low risk of bias for measurement of the outcome and 12 studies for missing outcome data domains. No study analysed data in accordance with a pre-published statistical analysis plan (SAP) causing all studies to be rated with some concerns for the risk of selection bias.

### Participant characteristics

The studies included 10,997 participants. Six studies focused exclusively on women [32, 36, 39, 40, 43, 48], the remaining 16 included both genders. One study did not provide a gender split [41] but the remaining 15 studies had an average of 55.6% female participants (range 2.98–81.7%).

Eight studies focused on specific ethnic groups or migrants from either Asia (Korea [37, 39], China [38], Vietnam or Indonesia [48]), or Mexico [30, 40, 42, 45]. All participants were adults and most studies covered the whole age spectrum however one study only included people aged 18–35 [32], two specified working age adults [38, 39], and six focused on the later years in life with minimum age of 40 [40], 50 [35], 55 [31, 52], 60 [44] and 65 [49] respectively.

### Health literacy definitions and measures

There was considerable variation in how health literacy was defined and measured (see Table 2). Six studies [36, 40, 42, 43, 45, 47] did not provide a definition, two [31, 34] gave a definition but no clear reference to identify it, and the remaining 14 studies referenced 10 different definitions (three studies [30, 35, 48] gave multiple definitions) with Ratzan and Parker [54] used in five studies [30, 35, 37, 39, 51] and Sorensen [6] cited in four studies [33, 48–50].

**Table 2:** Definitions and measures

1st author, year	Definition	Measure	Subjective /Objective	Culture specific	Condition specific
[30] Calderon, 2014	American Medical Association, 1999 [53], Ratzan and Parker, 2000 [54], World HealthOrganisation, 1998 [55]	Diabetes Health Literacy Scale [30]	Subjective		√
[50] Fiedler, 2019	Sorensen, 2012 [6]	German Health Literacy Questionnaire [56]	Subjective	√	
[32] Gharachourlo, 2018	Berkman, 2010 [57]	Iranian Health Literacy Questionnaire [58]	Subjective	√	
[39] Han, 2017	Ratzan and Parker, 2000 [54]	Assessment of Health Literacy in Cancer Screening [59]	Objective		√
[44] Liu, 2018	Mark, 2009 [60]	Chinese Citizen Health Literacy Questionnaire [61]	Subjective	√	
[42] Mas, 2017	None	STOFHLA [62]	Objective		
[45, 46] Mas, 2018 (and 2015)	None	TOFHLA [63]	Objective		
[38] Li, 2016	Baker, 2006 [64]	Malaria Health Literacy Questionnaire [38]	Unclear		√
[40] Otilingam, 2015	None	NVS [65]	Objective		
[47] Panahi, 2018	None	Health Literacy for Iranian Adults [66]	Subjective	√	
[43] Parekh, 2017	None	NVS [65]	Objective		
[31] Tai, 2016	Unclear	STOFHLA [62]	Objective		
[48] Tsai, 2018	Kimbrough, 2007 [67], Nutbeam, 2008 [2], Sorensen, 2012 [6]	Bespoke	Subjective		
[49] Uemura, 2018	Sorensen, 2012 [6]	HLS-EU-Q16 [68] and HLS-14 [69]	Subjective		
[41] Zhuang, 2016	American Medical Association [53]	Rapid Assessment of Health Literacy [41]	Objective		
[35] Banbury, 2019	Australian Bureau of statistics, 2006 [70] Berkman et al., 2011 [11] Ratzan and Parker, 2000 [54]	Health Literacy Questionnaire [71]	Subjective		
[36] Handa, 2020	None	HLS-14 [69]	Subjective		
[37] Kim, 2020	Ratzan and Parker, 2000 [54]	REALM, DM-REALM, TOFHLA, NVS [37, 63, 65, 72]	Objective		√ (DM-REALM)
[33] Knudsen, 2019	Sorensen, 2012 [6]	Health Literacy Questionnaire [71]	Subjective		
[51] McCaffery, 2019	Ratzan and Parker, 2000 [54]	Health Literacy Questionnaire [71]	Subjective		
[34] Tavakoly Sany, 2019	Unclear	TOFHLA [63]	Objective		
[52] Smith, 2019	Nutbeam, 2000 [2]	Health Literacy Questionnaire [71]	Subjective		

A variety of health literacy measures were used across the 22 studies. Four studies used condition specific measures [30, 37–39], four used culture specific measures [32, 44, 47, 50], and three created measures for use in the studies [30, 41, 48]. The rest used validated general instruments such as newest vital sign -NVS [37, 40, 43], test of functional health literacy in adults / short test of functional health literacy in adults -TOFHLA/STOFHLA [31, 34, 37, 42, 45], Health Literacy Questionnaire – HLQ [33, 35, 51, 52], HLS-EU-Q16 [49], and HLS14 [49]. Two studies used multiple measures [37, 49].

Studies did not always clearly describe measures used to assess health literacy. Nine studies used clearly objective measures [31, 34, 37, 39–43, 45], 12 used clearly subjective measures [30, 32, 33, 35, 36, 44, 47–52]. In addition one [38] was more difficult to determine and appears to be a mixed measure. The author has not responded to requests for clarification. With regards to the different aspects of health literacy (functional, interactive, critical) as shown in Table 3, all but one [48] measured functional health literacy, nine measured interactive health literacy [32, 33, 35, 36, 48–52] with a further two [38, 47] providing insufficient information to determine, and just six [32, 35, 36, 48, 49, 52] measured critical health literacy, with an additional three being unclear for this aspect [38, 47, 50].

**Table 3:** Aspects of Health Literacy covered by measure and intervention content

Reference, 1st author, year	Measure			Intervention			Measure/Intervention matches
	Functional	Interactive	Critical	Functional	Interactive	Critical	Insufficient/Matches/Exceeds
[30] Calderon, 2014	yes	no	no	yes	no	no	matches
[50] Fiedler, 2019	yes	yes	unclear	yes	yes	yes	insufficient
[32] Gharachourlo, 2018	yes	yes	yes	yes	yes	no	exceeds
[39] Han, 2017	yes	no	no	yes	yes	no	insufficient
[44] Liu, 2018	yes	no	no	yes	no	no	matches
[42] Mas,2017	yes	no	no	yes	unclear	no	insufficient
[45, 46] Mas, 2018 (and 2015)	yes	no	no	yes	yes	no	insufficient
[38] Li, 2016	yes	unclear	unclear	yes	yes	unclear	insufficient
[40] Otilingam, 2015	yes	no	no	yes	yes	no	insufficient
[47] Panahi, 2018	yes	unclear	unclear	yes	no	no	matches
[43] Parekh, 2017	yes	no	no	yes	yes	no	insufficient
[31] Tai, 2016	yes	no	no	yes	no	no	matches
[48] Tsai, 2018	no	yes	yes	yes	yes	yes	insufficient
[49] Uemura, 2018	yes	yes	yes	yes	yes	unclear	exceeds
[41] Zhuang, 2016	yes	no	no	yes	no	no	matches
[35] Banbury, 2020	yes	yes	yes	yes	yes	yes	matches
[36] Handa, 2020	yes	yes	yes	yes	yes	no	exceeds
[37] Kim, 2020	yes	no	no	yes	yes	no	insufficient
[33] Knudsen, 2019	yes	yes	no	yes	yes	no	matches
[51] McCaffery, 2019	yes	yes	no	yes	yes	unclear	insufficient
[34] Tavakoly Sany, 2019	yes	no	no	yes	yes	no	insufficient
[52] Smith, 2019	yes	yes	yes	yes	yes	yes	matches

### Intervention characteristics

All interventions targeted functional aspects of health literacy (see Table 3), in addition sixteen [32–40, 43, 45, 48–52] also targeted interactive aspects (with a further one [42] providing insufficient information to determine) and four of these [35, 48, 50, 52] also targeted critical health literacy (with a further three [38, 49, 51] being unclear). Intervention designs (as shown in Table 1) included small group sessions [32, 34, 35, 37, 39, 40, 42–45, 48–51], text or social media messages [38, 41, 47], animation [30], multi-media learning [52], app [36] and one to one education [31, 33]. The most common approach was for small group educational classes (14 studies).

Of the text/social media interventions the frequency of messages ranged from 3x/week for 4 months [38] through to once a week for a year [41]. One study used social media for health education counselling for a total of 6 sessions [47], but there was insufficient detail to identify length, duration or content of the sessions.

The 14 small group studies ranged from 40 min [44] to full day sessions [50], with a frequency ranging between twice a week [51], weekly [32, 35, 37, 40, 42, 45, 49] fortnightly [43, 48] and monthly [44]. The intervention duration period ranged from 2 weeks [40] to 12 months [37] of active content. One study did not specify frequency or total intervention duration, just individual session lengths [34]. Across all studies follow-up ranged from the same day [30] to 12 months [37, 41, 50]. The time lag between intervention end and follow up ranged from none [30, 36–38, 41–43, 45, 49] to 6 months [39, 48, 50].

### Intervention effects on main outcomes

Table 4 summarises the effect of intervention on both health literacy and behavioural outcomes.

**Table 4:** Summary of health literacy and behavioural outcome results

Reference, 1st author, year, country	Health Literacy		Behavioural outcomes		
	Significant result found?	Summary result	Behavioural outcome measured	Significant result found?	Summary result
**Domain**	**Health Care**				
[30] Calderon, 2014 USA	Yes	I > C: p = 0.03	None		
[31] Tai, 2016 USA	Yes	After adjusting for pre-score, I > C: p = 0.011	None		
[32] Gharachourlo, 2018 Iran	Yes	I > C: p < 0.001	10 dimensions of health, physical health, sports and fitness, weight management and nutrition, disease prevention, mental health, spiritual health, social health, avoidance of drugs, alcohol and opiates, accident prevention and environmental health.	Yes for 8/10 dimensions (not environmental health and spiritual health)	I > C: p < 0.001 (Overall lifestyle, physical health, sports & fitness, weight management & nutrition, disease prevention, mental health, social health, avoidance of drugs, alcohol and opiates, accident prevention)
[33] Knudsen, 2019 Denmark	For HLQ6 only	HLQ6: I > C p = 0.003	None		
[34] Tavakoly Sany, 2019 Iran	Yes	I > C: p < 0.05	Self-care behaviours for heart failure	Yes	I > C: p < 0.05
[35] Banbury, 2020 Australia	No	I vs C: p > 0.05	None		
[36] Handa, 2020 Japan	No	I vs C: p > 0.05	None		
[37] Kim. 2020 USA	Yes	REALM: I > C: 3, 6 months p < 0.01, 12 months p < 0.001 DM-REALM: I > C: 3,6,12 months p < 0.001 TOFHLA: I > C: 3 months p < 0.05 NVS: I > C: 3,6,12 months p < 0.05	None		
**Domain**	**Disease prevention**				
[38] Li 2016, Niger	Yes	I > C: p < 0.01	None		
[39] Han, 2017 USA	Yes	HL change T1 to T2 I > C (p < .05)	Self-reported mammogram at baseline and medical record review at post-test	Yes	I > C, OR 18.5 (95% confidence interval [CI] = 9.2, 37.4)
			Self-reported pap test at baseline and medical record review at post-test	Yes	I > C, OR 13.3 (95% CI = 7.9, 22.3)
			Self-reported mammogram and pap test at baseline and medical record review at post-test	Yes	I > C, OR 17.4 (95% CI = 7.5, 40.3)
**Domain**	**Health promotion**				
[40] Otilingam 2015, USA	Yes	(Combined intervention groups) I > C: p = 0.0103. HL change T1 to T2 I > C: 0.039 HL change T1 to T3 n.s	Behaviours to reduce dietary fats	Yes	T1 to T3 I > C: p = 0.0140.
[41] Zhuang, 2016 China	Yes	I > C: p < 0.001	None		
[42] Mas, 2017 USA	No	I vs C: p > 0.05	None		
[43] Parekh, 2017 USA	No	I vs C: p > 0.05	Nutrition literacy	No	I vs C: p > 0.05
			Fruit and vegetable intake	No	I vs C: p > 0.05
[44] Liu, 2018 China	Yes	I > C: p < 0.05	None		
[45, 46] Mas, 2018 (and 2015) USA	Yes	Change score I > C: p = 0.01	34 item questionnaire measuring nutrition & physical activity behaviours	Yes, in adjusted model	I > C: p = 0.049.
[47] Panahi, 2018 Iran	Yes	HL change T1 –T2 -T3 I > C: p = 0.014	Smoking behaviour	Yes	I > C: p < 0.0001
[48] Tsai, 2018 Taiwan	No	I vs C: p > 0.05	None		
[49] Uemura, 2018 Japan	For some but not all domains	HLS-EU-Q16 I > C: disease prevention score p = 0.04 HLS-14 I > C: Total score p = 0.03, Communicative score p = 0.01, critical score, p = 0.02	Dietary habits – food frequency	Yes	I > C p = 0.001
			Dietary habits – dietary variety	Yes	I > C p = 0.04
			Steps per day	Yes	I > C: p < 0.001
			Total energy expenditure (physical activity level x basal metabolic rate)	Yes	I > C: p = 0.01
[50] Fiedler, 2019 Germany	Mixed and inconclusive	A significant effect was only seen for proactive help for the intervention group at T1.	None		
[51] McCaffery 2019, Australia	HLQ3 only	HLQ3: I > C p = 0.01	None		
[52] Smith, 2019 Australia	No	I vs C: p > 0.05	None		

Note: *I* Intervention, *C* Control

#### Health literacy

Twelve of the studies showed a significant increase in health literacy in the intervention group compared to the control group [30–32, 34, 37–41, 44, 45, 47]. Six showed no significant difference [35, 36, 42, 43, 48, 52], three showed an increase in health literacy for some but not all domains or subscales [33, 49, 51] and one was inconclusive due to mixed results in a crossover design [50]. Four out of the six with no change employed subjective measures [35, 36, 48, 52].

#### Behavioural outcomes

Many of the studies included additional outcomes such as knowledge [39, 40, 47], self-efficacy [34, 37, 52] morbidity [38], perceptions [39, 47], physical and cognitive function [49], health education impact [35], patient activation [33] and behavioural outcomes [32, 34, 39, 40, 43, 45, 47, 49]. Behaviour was measured in smoking prevention behaviours [47], nutrition related behaviours [40, 43, 49], physical activity behaviours [49], cancer screening behaviours [39], and some measures which encompassed a variety of domains (lifestyle [32], self-care [34] and cardiovascular health [45]).

Smoking prevention behaviours, physical activity and cancer screening were measured in a single study each, and all showed significant changes in favour of the intervention group [39, 47, 49]. Nutrition and diet related behaviours were measured in three studies. Two [40, 49] showed significant results in favour of the intervention group (fat related diet habits, food frequency and dietary variety). The third study [43] measured nutrition literacy and fruit and vegetable intake and found no significant effect of intervention.

Lifestyle factors were measured in one study [32]. This measure considered 10 dimensions of health and found a statistically significant effect of the intervention for overall lifestyle, and for 8 out of 10 sub-dimensions. Cardiovascular health was measured in one study [45] by measuring nutrition and physical activity behaviours and found a significant change in intervention group compared to control. Finally one study [34] found a significant change in intervention compared to control for self-care behaviours in heart failure patients.

#### Cardiac patients

Two studies focused on cardiac patients [33, 34] and a further two of the studies used a cardiovascular health curriculum within healthy adults to reduce the risk of cardiovascular disease [40, 45]. Tavakoly Sany [34] focused on heart failure patients and ran three educational group workshops using techniques such as teach back and role playing. The study measured health literacy, self-efficacy and self-care behaviours and found a significant effect of intervention in all three aspects – both immediately post intervention and at the three month follow up. Knudsen [33] compared tele-rehabilitation with usual care cardiac rehabilitation for both health literacy and patient activation. They found that neither method of rehabilitation improved patient activation and only one of the HLQ subscales (ability to engage with healthcare providers) showed a significant effect in the intervention group.

Two studies focused on reducing cardiovascular risk in health adults. Both studies were conducted in America and targeted Spanish speaking immigrants. Mas [45] used a combined health literacy and standard English as a second language (ESL) curriculum which used “Salud para su Corazon” (health for your heart) as the main resource. The study measured both health literacy and cardiovascular health behaviours and found a significant effect of intervention in both (although they were not correlated). Otilingam [40] used specifically designed content in two 2-h workshops designed to improve heart health and brain health in Latina’s. The paper measured health literacy and dietary fat reduction behaviours and found significant effects of intervention in both.

## Discussion

This systematic review included 22 studies from nine countries involving almost 11,000 participants. In 68% of studies a significant improvement in health literacy was seen. Additionally, eight studies measured behavioural outcomes and in seven of the studies a significant effect in favour of the intervention group was found. Only two studies have been carried out with cardiovascular patients.

Quality appraisal found that no studies were at low risk of bias. This was largely influenced by the lack of pre-published SAP protocols and issues with effective randomisation, allocation concealment and blinding which can be more challenging in this type of interventional study, though not impossible [73]. Analysis of intervention reporting showed that studies were generally poor at reporting sufficient detail of the intervention content to allow replication (and in some cases, effective categorisation of intervention focus).

Notwithstanding this, this systematic review has highlighted the growth in the health literacy field. This review set no lower limit date yet the oldest study including a pre-post measure of health literacy in a controlled trial was 2014. The number of studies has steadily increased (with over half being published since 2018) suggesting a growth in work to establish the evidence base for health literacy interventions. Whilst the most commonly used approach was small group educational interventions it is worth noting that other methods that are less time/resource intensive show promise. A short animation [30], a single 10 min training session [31], remote videoconferencing/tele-rehabilitation [33, 35] and three studies that used social media or SMS messages [38, 41, 47] were all effective at increasing health literacy.

The search for this systematic review captured studies that were not available when the review of community-based interventions was conducted by Nutbeam et al. in 2018 [74]. As Nutbeam [74] indicated, there is a move towards the inclusion of wider aspects of health literacy, with 16 of the interventions in the current review now clearly including interactive aspects but only four interventions clearly including aspects of critical health literacy. It is promising to see more interactive content, but there is an evident lag in including aspects of critical health literacy. This could reflect difficulties in operationalising critical health literacy in measures. For example, a recent study into health literacy interventions in Europe [75] found seven studies (not eligible for this systematic review) with critical health literacy content, but in common with this review, only three of the interventions included any form of critical health literacy measure, and even then it was measured via skills lists, interview or decision-making skills rather than specific health literacy instruments.

This mismatch between measures and intervention content can have significant effects. Half of the studies in this review did not have measures capable of measuring all aspects of health literacy targeted in the intervention [34, 37–40, 42, 43, 45, 48, 50, 51]. Notably four out of the seven studies that did not find an increase in health literacy as a result of the intervention fell within this group [42, 43, 48, 50]. In addition, only 12 of the studies [35, 37, 38, 40, 41, 43, 45, 47–49, 51, 52] included an indication of their intervention’s theoretical underpinnings. Whilst there is debate as to whether theory contributes to the efficacy of interventions [76, 77], in a field which is striving to develop an evidence base, theory allows for the systematic development, comparison and refinement of interventions and is something that should be encouraged [78].

As a determinant of health, health literacy may offer a way to improve outcomes for populations most at risk of health inequalities. Whilst several studies focus on migrants, other at-risk populations have not been similarly targeted for intervention. This is particularly noticeable with regards to gender. Recent studies have suggested men have lower health literacy than women [79–81], and are more likely to have multiple lifestyle risks [68, 82, 83] yet from our pool of 22 studies six focused exclusively on women and no study focused solely on men.

Only two studies (both within the past year) have been conducted with cardiac patients. One of these compared two different modes of cardiac rehabilitation, and the other conducted three education sessions with heart failure patients. It is surprising that more interventions have not been conducted with cardiac patients as yet, given that evidence shows they are at higher risk for health literacy issues [14, 15] and that they can benefit from behavioural interventions. The evidence in this review suggests that health literacy interventions are effective at influencing behaviour, though as no study conducted mediation analysis, we are unable to confirm the direction of this influence.

This review has highlighted rapid growth in intervention studies with just five studies published up until 2016 and then a rapid increase with three in 2017, six in 2018, five in 2019 and three in the first quarter of 2020. We can also see evidence of improved methodological designs in later studies – perhaps as a result of comments by Brainard et al. [84] regarding methodological challenges in health literacy research. A key observation was that there is not enough focus on patient-centred outcomes, and interventions could be more useful if they involved patients in the design - rather than assuming that simply telling people what they need to do is sufficient to bring about change. Three studies [34, 35, 37] in 2019 and 2020 involved participants in intervention content and design. In addition, the only four studies [34, 36, 37, 52] with lower risk of bias (some concerns) were from 2019 to 2020.

The ultimate aim of health literacy interventions is to bring about behaviour change in order to make an individual/agent/organisation behave in a health literate way. In recent years we can see the behaviour change field has developed a considerable number of frameworks, theories, components and techniques. Whilst this review started out asking if health literacy interventions influenced behaviour change, it has become apparent that heath literacy interventions can both influence behaviour change but could also learn from behaviour change theory. Interventions designed along behaviour change principles have the potential to be more robust, effective and applicable – at all levels.

### Limitations

This review is the first (to the best of our knowledge) to focus on controlled trial health literacy interventions with pre and post measures, in adults across all health conditions and domains. It adds to the body of knowledge by demonstrating that controlled trial health literacy interventions are increasing rapidly and can be an effective method of both improving health literacy and changing health behaviours. Nevertheless, there are limitations. It is known that there are close links between the concept of health literacy and other concepts such as activation, empowerment and education. By restricting the search terms to studies which identify themselves as health literacy interventions and include a pre-post health literacy measure it is possible we have missed other studies which may have demonstrated effects on health literacy. In addition, we have restricted the search to full peer reviewed and published English language quantitative papers only, and there may well be qualitative studies, or studies in other languages that can contribute to the review question. In addition, it should be noted that all included studies were at risk of bias with 18 judged at high risk. This may impact on ability to draw reliable conclusions from the included studies.

Given health literacy has been around as a concept with dedicated measures for over 30 years it is essential that health literacy begins to operate as a clearly defined concept, with its own terms, measures and dedicated interventions. By restricting the search terms to health literacy specific studies, we begin to demarcate the field and strengthen the evidence base for health literacy interventions.

### Conclusions

Even allowing for the strict inclusion criteria applied, 22 studies were found with health literacy interventions. Fifteen of these studies demonstrated that interventions targeting health literacy can improve health literacy. In addition, seven out of eight studies with a behavioural outcome found that the health literacy intervention had a significant effect on behaviour. The health literacy field is growing rapidly, with all studies published since 2014 and over half since 2018. In order to continue to develop the evidence base, health literacy interventions should begin to consider the wider aspects of health literacy and make better use of behaviour change theory to more effectively change the health literacy behaviour of participants – which in turn may help behaviour change interventions be more effective.

## Supplementary information

**Additional file 1: Supplementary Table 1.** PRISMA-S, search results, screening decisions and search strategies.

**Additional file 2: Supplementary Table 2.** Coding for the 12 TIDieR items for individual studies, divided into intervention and control conditions.

**Additional file 3: Supplementary Table 3.** Percentage of studies scoring at each reporting grade for the 12 TIDieR items for individual studies, divided into intervention and control conditions.

**Additional file 4: Supplementary Table 4.** Risk of bias.

## Data Availability

The data that support the findings of this study are available from the corresponding author upon reasonable request.

## Abbreviations

ESL: English as a second language
HLQ: Health literacy questionnaire
HRQOL: Health related quality of life
NHS: National health service
NVS: Newest vital sign
PRISMA: Preferred reporting items for systematic reviews and meta-analyses
RCT: Randomised controlled trial
RoB 2: Risk of Bias 2
ROBINS-I: Risk of bias in non-randomised studies of interventions
SAP: Statistical analysis plan
STOFHLA: Short test of functional health literacy in adults
TIDieR: Template for intervention description and replication
TOFHLA: Test of functional health literacy in adults
