# Supplementary material for: Establishing the efficacy of interventions to improve health literacy and health behaviours: a systematic review
Source: BMC Public Health. 2020 Jun 30;20:1040. doi: 10.1186/s12889-020-08991-0 (PMC7329558; doi:10.1186/s12889-020-08991-0)
Supplement: Supplementary file 1 — Additional file 1: Supplementary Table 1. PRISMA-S, search results, screening decisions and search strategies. [file 12889_2020_8991_MOESM1_ESM.docx]

Supplementary table 1: PRISMA-S, search results, screening decisions and search strategies.

**PRISMA-S reporting checklist.**

| **Section/topic** | **#** | **Checklist item** | **Location** |
| --- | --- | --- | --- |
| **INFORMATION SOURCES AND METHODS** | | | |
| Database name | 1 | Name each individual database searched and state the platform for each individual database searched. | Methods,  Supplementary table 1 |
| Multi-database Searching | 2 | If databases were searched simultaneously through a single interface, state the name of the interface and list all of the databases included and their dates of coverage individually. | n/a |
| Study registries | 3 | List any study registries searched. | n/a |
| Online resources and browsing | 4 | Describe any online or print source purposefully searched or browsed, and described any methods used (e.g., table of contents, print conference proceedings, web sites). | n/a |
| Citation searching | 5 | Indicate whether cited references or citing references were examined, and describe any methods used for locating cited/citing references (e.g., browsing reference lists, name and platform for any citation index used, email alerts). | Methods,  Supplementary table 1 |
| Contacts | 6 | Indicate whether additional studies or data were sought by contacting authors, experts, manufacturers, or other contacts. | n/a |
| Other methods | 7 | Describe any additional information sources or search methods used. | n/a |
| **SEARCH STRATEGIES** | | | |
| Full search strategies | 8 | Include the search strategies for each database and resource, copied and pasted exactly as run, including any updates. | Supplementary table 1 |
| Limits and restrictions | 9 | Specify that no limits were used or describe any limits or restrictions applied to a search and provide justification for their use (e.g., date or time period, language, study design). | Supplementary table 1 |
| Search filters | 10 | Indicate and cite when published search filters or hedges were used for any search, and whether they were modified or adapted from their published versions. | Supplementary table 1 |
| Prior work | 11 | Indicate and cite when search strategies from other literature reviews were adapted or reused for part or all of the search. | n/a |
| Updates | 12 | Report the methods used to update the search(es) (e.g., rerunning searches, email alerts). | Supplementary table 1 |
| Dates of searches | 13 | For each search strategy, provide the date when the search occurred. | Supplementary table 1 |
| **PEER REVIEW** | | | |
| Peer review | 14 | Describe any search peer review process. | Methods |
| **MANAGING RECORDS** | | | |
| Total records | 15 | Document the total number of references identified from each database and additional information source. | Supplementary table 1 |
| Deduplication | 16 | Describe the processes and any software used to deduplicate records from multiple database or other resource searches. | Methods |

Preferred Reporting Items for Systematic review and Meta-Analysis Searches (PRISMA-S) 2019 statement

Rethlefsen ML, Koffel JB, Kirtley S, Waffenschmidt S, Ayala AP, PRISMA-S Group.

Version 2.3, released December 6, 2019.

**Search Results**

| **Platform** | **Database** | **Results** |
| --- | --- | --- |
| **EBSCO** | CINAHL | 237 |
|  | Psych & behavioural sciences collection | 17 |
| **OVID** | Embase | 621 |
|  | Medline | 434 |
|  | ERIC | 14 |
|  | Psychinfo (1967-present) | 203 |
|  | HMIC | 1175 |
|  | NHS Scotland Journals | 41 |
|  | Social policy and practice | 5 |
|  | Global Health | 80 |
| Web of Science | | 374 |
| Scopus | | 677 |
| Social care online | | 9 |
| **Total** | | **3387** |

**Searches run between May 2018 and April 2020.**

**Screening decisions**

**Title/abstract screening decisions**

| **Reasons** | **Results** |
| --- | --- |
| Total | 3387 |
| De-Duplicate | 1760 |
| Wrong population | 321 |
| Scale development | 202 |
| Review | 124 |
| Background | 40 |
| No intervention | 1189 |
| No HL measure | 28 |
| No post measure | 102 |
| No pre-measure | 9 |
| Not HL | 14 |
| No control | 21 |
| Protocol | 16 |
| Abstracts only | 2 |
| Unpublished thesis | 2 |
| Full paper not available/no response from authors | 6 |
| **Included for full text screening:** | **51** |
| Add: texts found from reference searches | 6 |
| **TOTAL** | **57** |

**Full text decisions**

| **Reasons** |  |
| --- | --- |
| Total for full screening | 57 |
| Protocol | 2 |
| No HL measure | 2 |
| No pre-measure | 2 |
| No post measure | 13 |
| Conference abstract | 3 |
| Not HL intervention | 2 |
| Same data set as another paper in the study | 1 |
| No control | 10 |
| **Included** | **22** |

**Search strategies.**

**EBSCO (CINAHL, Psychology and behavioural sciences collection)**

| S1 | MH (“Health Literacy”) |
| --- | --- |
| S2 | AB health liter* OR TI = health liter* |
| S3 | AB functional health liter* OR TI = functional health liter* |
| S4 | AB interactive health liter* OR TI = interactive health liter* |
| S5 | AB communicati* health liter* OR TI = communicati* health liter* |
| S6 | AB critical health liter* OR TI = critical health liter* |
| S7 | S1 OR S2 OR S3 OR S4 OR S5 OR S6 |
| S8 | AB intervention* or TI intervention* |
| S9 | AB trial* or TI trial* |
| S10 | AB (control or controlled) OR TI (control or controlled) |
| S11 | AB pre-post or TI pre-post |
| S12 | AB experiment* or TI experiment* |
| S13 | S8 OR S9 OR S10 OR S11 OR S12 |
| S14 | AB (health liter* screen* or screen* health liter*) OR TI (health liter* screen* or screen* health liter*) |
| S15 | AB (health liter* measure* or measure* health liter*) OR TI (health liter* measure* or measure* health liter*) |
| S16 | AB (health liter* survey* OR survey* health liter*) OR TI (health liter* survey* OR survey* health liter*) |
| S17 | AB rapid estimate of adult literacy in medicine OR TI rapid estimate of adult literacy in medicine |
| S18 | AB test of functional health literacy OR TI test of functional health literacy |
| S19 | AB medical term recognition test OR TI medical term recognition test |
| S20 | AB short assessment of health literacy OR TI short assessment of health literacy |
| S21 | AB health literacy questionnaire OR TI health literacy questionnaire |
| S22 | AB newest vital sign OR TI newest vital sign |
| S23 | AB health activities literacy scale OR TI health activities literacy scale |
| S24 | AB conversational health literacy assessment tool OR TI conversational health literacy assessment tool |
| S25 | AB HLS-EU-Q47 OR TI HLS-EU-Q47 |
| S26 | AB HLS-EU-Q16 OR TI HLS-EU-Q16 |
| S27 | AB HL-SF12 OR TI HL-SF12 |
| S28 | AB HLS-Q12 OR TI HLS-Q12 |
| S29 | S14 OR S15 OR S16 OR S17 OR S18 OR S19 OR S20 OR S21 OR S22 OR S23 OR S24 OR S25 OR S26 OR S27 OR S28 |
| S30 | S7 AND S13 AND S29 |

**OVID (Medline, Embase, ERIC, Psychinfo (1967-present), HMIC, NHS Scotland Journals, Social policy and practice, Global health)**

| 1 | health literacy/ |
| --- | --- |
| 2 | health liter*.ab. or health liter*.ti. |
| 3 | functional health liter*.ab. or functional health liter*.ti. |
| 4 | interactive health liter*.ab or interactive health liter*.ti. |
| 5 | communicati* health liter*.ab or communicati* health liter*.ti. |
| 6 | critical health liter*.ab or critical health liter*.ti. |
| 7 | 1 or 2 or 3 or 4 or 5 or 6 |
| 8 | intervention*.ab. or intervention*.ti. |
| 9 | trial*.ab. or trial*.ti. |
| 10 | (control or controlled).ab. or (control or controlled).ti. |
| 11 | pre-post.ab. or pre-post.ti. |
| 12 | experiment*.ab. or experiment*.ti. |
| 13 | 8 or 9 or 10 or 11 or 12 |
| 14 | (health liter* screen* or screen* health liter*).ab. or (health liter* screen* or screen* health liter*).ti. |
| 15 | (health liter* measure* or measure* health liter*).ab. or (health liter* measure* or measure* health liter*).ti. |
| 16 | (health liter* survey* OR survey* health liter*).ab. or (health liter* survey* OR survey* health liter*).ti. |
| 17 | rapid estimate of adult literacy in medicine.ab. or rapid estimate of adult literacy in medicine.ti. |
| 18 | test of functional health literacy.ab.or test of functional health literacy.ti. |
| 19 | medical term recognition test.ab. or medical term recognition test.ti. |
| 20 | short assessment of health literacy.ab. or short assessment of health literacy.ti. |
| 21 | health literacy questionnaire.ab. or health literacy questionnaire.ti. |
| 22 | newest vital sign.ab. or newest vital sign.ti. |
| 23 | health activities literacy scale.ab. or health activities literacy scale.ti. |
| 24 | conversational health literacy assessment tool.ab. or conversational health literacy assessment tool.ti. |
| 25 | HLS-EU-Q47.ab. or HLS-EU-Q47.ti. |
| 26 | HLS-EU-Q16.ab. or HLS-EU-Q16.ti. |
| 27 | HL-SF12.ab. or HL-SF12.ti. |
| 28 | HLS-Q12.ab. or HLS-Q12.ti. |
| 29 | 14 or 15 or 16 or 17 or 18 or 19 or 20 or 21 or 22 or 23 or 24 or 25 or 26 or 27 or 28 |
| 30 | 7 and 13 and 29 |

**SCOPUS**

| 1 | TITLE-ABS-KEY (“HEALTH LITERACY”) |
| --- | --- |
| 2 | TITLE-ABS-KEY (HEALTH LITER*”) |
| 3 | TITLE-ABS-KEY (“FUNCTIONAL HEALTH LITER*”) |
| 4 | TITLE-ABS-KEY (“INTERACTIVE HEALTH LITER*”) |
| 5 | TITLE-ABS-KEY (“COMMUNICATI* HEALTH LITER*”) |
| 6 | TITLE-ABS-KEY (“CRITICAL HEALTH LITER*”) |
| 7 | #1 OR #2 OR #3 OR #4 OR #5 OR #6 |
| 8 | TITLE-ABS-KEY (INTERVENTION*) |
| 9 | TITLE-ABS-KEY (TRIAL*) |
| 10 | TITLE-ABS-KEY (CONTROL OR CONTROLLED) |
| 11 | TITLE-ABS-KEY (“PRE-POST”) |
| 12 | TITLE-ABS-KEY (EXPERIMENT*) |
| 13 | #8 OR #9 OR #10 OR #11 OR #12 |
| 14 | TITLE-ABS-KEY (“HEALTH LITER* SCREEN*” OR “SCREEN* HEALTH LITER*”) |
| 15 | TITLE-ABS-KEY (“HEALTH LITER* MEASURE*” OR “MEASURE* HEALTH LITER*”) |
| 16 | TITLE-ABS-KEY (“HEALTH LITER* SURVEY” OR “SURVEY HEALTH LITER*”) |
| 17 | TITLE-ABS-KEY (“RAPID ESTIMATE OF ADULT LITERACY IN MEDICINE”) |
| 18 | TITLE-ABS-KEY (“TEST OF FUNCTIONAL HEALTH LITERACY”) |
| 19 | TITLE-ABS-KEY (“MEDICAL TERM RECOGNITION TEST”) |
| 20 | TITLE-ABS-KEY (“SHORT ASSESSMENT OF HEALTH LITERACY”) |
| 21 | TITLE-ABS-KEY (“HEALTH LITERACY QUESTIONNAIRE”) |
| 22 | TITLE-ABS-KEY (“NEWEST VITAL SIGN”) |
| 23 | TITLE-ABS-KEY (“HEALTH ACTIVITIES LITERACY SCALE”) |
| 24 | TITLE-ABS-KEY (“CONVERSATIONAL HEALTH LITERACY ASSESSMENT TOOL”) |
| 25 | TITLE-ABS-KEY (“HLS-EU-Q47”) |
| 26 | TITLE-ABS-KEY (“HLS-EU-Q16”) |
| 27 | TITLE-ABS-KEY (“HL-SF12”) |
| 28 | TITLE-ABS-KEY (“HLS-Q12”) |
| 29 | #14 OR #15 OR #16 OR #17 OR #18 OR #19 OR #20 OR #21 OR #22 OR #23 OR #24 OR #25 OR #26 OR #27 OR #28 |
| 30 | #7 AND #13 AND #29 |

**Social care online**

| 1 | AllFields ‘health literacy’ |
| --- | --- |
| 2 | Publication Title: ‘functional health literacy’ OR AbstractOmitNorms: ‘functional health literacy’ |
| 3 | Publication Title: ‘interactive health literacy’ OR AbstractOmitNorms: ‘interactive health literacy’ |
| 4 | Publication Title: ‘communicative health literacy’ OR AbstractOmitNorms: ‘communicative health literacy’ |
| 5 | Publication Title: ‘critical health literacy’ OR AbstractOmitNorms: ‘critical health literacy’ |
| 6 | 1 or 2 or 3 or 4 or 5 |
| 7 | Publication title: ‘intervention’ OR AbstractOmitNorms: ‘intervention’ |
| 8 | Publication title: ‘Control or controlled’ OR AbstractOmitNorms: ‘control or controlled’ |
| 9 | Publication title: ‘“pre-post”’ OR AbstractOmitNorms: ‘”pre-post”’ |
| 10 | Publication title: ‘Experiment’ OR AbstractOmitNorms: ‘experiment’ |
| 11 | 7 or 8 or 9 or 10 |
| 12 | 6 AND 11 |

**Web of Science**

| #1 | TI = health liter* |
| --- | --- |
| #2 | TI = functional health liter* |
| #3 | TI = interactive health liter* |
| #4 | TI = communicate* health liter* |
| #5 | TI = critical health liter* |
| #6 | #1 OR #2 OR #3 OR #4 OR #5 |
| #7 | TS = (health AND literacy AND measure) |
| #8 | TS = (health AND literacy AND survey) |
| #9 | TS = (health AND literacy AND screen*) |
| #10 | #7 OR #8 OR #9 |
| #11 | TI=intervention |
| #12 | #6 AND #10 AND #11 |
