# Supplementary material for: Establishing the efficacy of interventions to improve health literacy and health behaviours: a systematic review
Source: BMC Public Health. 2020 Jun 30;20:1040. doi: 10.1186/s12889-020-08991-0 (PMC7329558; doi:10.1186/s12889-020-08991-0)
Supplement: Supplementary file 2 — Additional file 2: Supplementary Table 2. Coding for the 12 TIDieR items for individual studies, divided into intervention and control conditions. [file 12889_2020_8991_MOESM2_ESM.docx]

Supplementary Table 2 – Coding for the 12 TIDieR items for individual studies, divided into intervention and control conditions.

| Study | Condition | Item 1 | Item 2 | Item 3 | Item 4 | Item 5 | Item 6 | Item 7 | Item 8 | Item 9 | Item 10 | Item 11 | Item 12 |
| --- | --- | --- | --- | --- | --- | --- | --- | --- | --- | --- | --- | --- | --- |
| Calderon (2014) | Intervention | Yes | Yes | Yes | No | No | No | Unclear | Unclear | n/a | n/a | No | No |
|  | Control | Yes | Yes | Yes | Yes | No | Unclear | Unclear | Yes | n/a | n/a | No | No |
| Han (2016) | Intervention | Yes | Yes | Unclear | Yes | Yes | Yes | Yes | Yes | Unclear | n/a | Unclear | No |
|  | Control | Yes | Yes | No | Yes | No | Yes | Yes | Yes | n/a | n/a | No | No |
| Li (2016) | Intervention | Yes | Yes | Unclear | Yes | Yes | Yes | Yes | Yes | n/a | n/a | Unclear | No |
|  | Control | Yes | Yes | No | Yes | No | Yes | Yes | Yes | n/a | n/a | No | No |
| Gharachourlo (2018) | Intervention | Yes | Yes | Yes | Unclear | No | Yes | No | Yes | n/a | n/a | No | No |
|  | Control | Yes | Yes | Unclear | Yes | No | Yes | No | Yes | n/a | n/a | No | No |
| Liu (2018) | Intervention | Yes | Yes | Unclear | Yes | Yes | Yes | No | Yes | n/a | n/a | No | No |
|  | Control | Yes | Yes | Unclear | Yes | Yes | Yes | Unclear | Yes | n/a | n/a | No | No |
| Mas (2017) | Intervention | Yes | Yes | Unclear | Unclear | Yes | Yes | Unclear | Yes | n/a | m/a | No | No |
|  | Control | Yes | Yes | Yes | Yes | Unclear | Yes | Unclear | Yes | n/a | n/a | No | No |
| Mas (2018) | Intervention | Yes | Yes | Yes | Yes | Unclear | Yes | No | Yes | n/a | n/a | Yes | no |
|  | Control | Yes | Yes | Yes | Yes | Yes | Yes | No | Yes | n/a | n/a | Yes | no |
| Panahi (2017) | Intervention | Yes | Yes | No | Unclear | No | Yes | Yes | Unclear | n/a | n/a | No | no |
|  | Control | Yes | Yes | n/a | n/a | n/a | n/a | n/a | n/a | n/a | n/a | n/a | n/a |
| Parekh (2017) | Intervention | Yes | Yes | Yes | Yes | Yes | Yes | Unclear | Yes | n/a | n/a | No | No |
|  | Control | Yes | Yes | Yes | Yes | No | Yes | n/a | Yes | n/a | n/a | n/a | n/a |
| Tai (2016) | Intervention | No | Yes | Yes | Yes | Yes | Yes | Yes | Yes | No | n/a | No | No |
|  | Control | No | Yes | n/a | n/a | n/a | n/a | n/a | n/a | n/a | n/a | n/a | n/a |
| Tsai (2018) | Intervention | yes | yes | Yes | yes | yes | yes | No | Yes | n/a | n/a | Yes | Unclear |
|  | Control | Yes | Yes | n/a | n/a | n/a | n/a | n/a | n/a | n/a | n/a | n/a | n/a |
| Uemura (2018) | Intervention | Yes | Yes | Unclear | Yes | Yes | Yes | No | Yes | n/a | n/a | No | No |
|  | Control | Yes | Yes | n/a | n/a | n/a | n/a | n/a | n/a | n/a | n/a | n/a | n/a |
| Zhuang (2016) | Intervention | Yes | Yes | Unclear | Unclear | No | Unclear | No | Unclear | n/a | n/a | No | No |
|  | Control | Yes | Yes | No | Unclear | No | No | Unclear | No | n/a | n/a | No | No |
| Fielder (2018) | Intervention | Yes | Yes | Yes | Unclear | Unclear | Yes | Unclear | Yes | n/a | n/a | No | No |
|  | Control | Yes | Yes | Yes | Unclear | Unclear | Yes | Unclear | yes | n/a | n/a | No | No |
| Otilingam (2015) | Intervention | Yes | Yes | Yes | Yes | Yes | Yes | Yes | Yes | n/a | n/a | Unclear | No |
|  | Control | Yes | Yes | n/a | yes | n/a | n/a | n/a | n/a | n/a | n/a | n/a | n/a |
| Banbury (2020) | Intervention | Yes | Yes | Unclear | Yes | Unclear | Yes | Yes | Yes | Yes | Yes | No | No |
|  | Control | Yes | Yes | No | No | No | No | No | No | No | n/a | No | No |
| Handa (2020) | Intervention | Yes | Yes | Unclear | Yes | No | Yes | Yes | Yes | n/a | n/a | Yes | Yes |
|  | Control | Yes | Yes | Yes | Yes | No | Yes | Yes | n/a | n/a | n/a | No | No |
| Kim (2020) | Intervention | Yes | Yes | Unclear | Yes | Unclear | Yes | Yes | Yes | n/a | n/a | Yes | No |
|  | Control | Yes | Yes | Unclear | n/a | n/a | n/a | n/a | n/a | n/a | n/a | n/a | n/a |
| Knudsen (2019) | Intervention | Yes | Yes | Unclear | Yes | Yes | Yes | Yes | Unclear | Yes | n/a | No | No |
|  | Control | Yes | Yes | Unclear | Yes | Yes | Yes | Yes | Unclear | n/a | n/a | No | No |
| McCaffery (2019) | Intervention | Yes | Yes | Unclear | No | Yes | Yes | Yes | Unclear | Unclear | n/a | No | No |
|  | Control | Yes | Yes | No | No | Yes | Yes | Yes | Unclear | No | n/a | No | No |
| Tavakoly Sany (2019) | Intervention | Yes | Yes | Yes | Unclear | Unclear | Unclear | Yes | Unclear | Unclear | n/a | No | no |
|  | Control | Yes | Yes | n/a | Unclear | Unclear | Unclear | Yes | Unclear | n/a | n/a | n/a | n/a |
| Smith (2019) | Intervention | Yes | Yes | Unclear | Yes | No | Yes | Yes | Yes | n/a | n/a | No | No |
|  | Control | Yes | Yes | Unclear | Yes | No | Yes | Yes | Yes | n/a | n/a | No | No |

**Note:** Yes – clear description of item; No – no description or minimal description of item; Unclear – unclear description of item; n/a – the design of the study voided the relevance of this item.

**Items**: 1) Brief name 2) Why 3) What 4) Procedures 5) Who 6) How 7) Where 8) When & how much 9) Tailoring 10) Modifications 11) Planned fidelity/adherence assessment 12) Actual fidelity/adherence assessment
