# Supplementary material for: Establishing the efficacy of interventions to improve health literacy and health behaviours: a systematic review
Source: BMC Public Health. 2020 Jun 30;20:1040. doi: 10.1186/s12889-020-08991-0 (PMC7329558; doi:10.1186/s12889-020-08991-0)
Supplement: Supplementary file 3 — Additional file 3: Supplementary Table 3. Percentage of studies scoring at each reporting grade for the 12 TIDieR items for individual studies, divided into intervention and control conditions. [file 12889_2020_8991_MOESM3_ESM.docx]

Supplementary Table 3 – Percentage of studies scoring at each reporting grade for the 12 TIDieR items for individual studies, divided into intervention and control conditions.

| Study | Condition | Item 1 | Item 2 | Item 3 | Item 4 | Item 5 | Item 6 | Item 7 | Item 8 | Item 9 | Item 10 | Item 11 | Item 12 |
| --- | --- | --- | --- | --- | --- | --- | --- | --- | --- | --- | --- | --- | --- |
| Yes (%) | Intervention | 95% | 100% | 41% | 64% | 50% | 86% | 55% | 73% | 33% | 100% | 18% | 5% |
|  | Control | 100% | 100% | 38% | 69% | 31% | 69% | 43% | 64% | 0% | 0% | 8% | 0% |
| Unclear (%) | Intervention | 0% | 0% | 55% | 27% | 63% | 9% | 18% | 27% | 50% | 0% | 14% | 5% |
|  | Control | 0% | 0% | 31% | 19% | 19% | 19% | 36% | 21% | 0% | 0% | 0% | 0% |
| No (%) | Intervention | 5% | 0% | 5% | 5% | 27% | 5% | 27% | 0% | 17% | 0% | 68% | 91% |
|  | Control | 0% | 0% | 31% | 13% | 50% | 13% | 21% | 14% | 100% | 0% | 92% | 100% |
| N/A (n of studies) | Intervention | 0 | 0 | 0 | 0 | 0 | 0 | 0 | 0 | 16 | 21 | 0 | 0 |
|  | Control | 0 | 0 | 6 | 6 | 6 | 5 | 8 | 6 | 20 | 22 | 9 | 9 |

**Note**: Yes – clear description of item; No – no description or minimal description of item; Unclear – unclear description of item; n/a – the design of the study voided the relevance of this item. Percentages are calculated using only studies for whom each item is applicable and may not add up to 100 due to rounding.

**Items**: 1) Brief name 2) Why 3) What 4) Procedures 5) Who 6) How 7) Where 8) When & how much 9) Tailoring 10) Modifications 11) Planned fidelity/adherence assessment 12) Actual fidelity/adherence assessment
