# Supplementary material for: Establishing the efficacy of interventions to improve health literacy and health behaviours: a systematic review
Source: BMC Public Health. 2020 Jun 30;20:1040. doi: 10.1186/s12889-020-08991-0 (PMC7329558; doi:10.1186/s12889-020-08991-0)
Supplement: Supplementary file 4 — Additional file 4: Supplementary Table 4. Risk of bias. [file 12889_2020_8991_MOESM4_ESM.docx]

| Supplementary Table 4 – Risk of bias | | | | | | | | | |
| --- | --- | --- | --- | --- | --- | --- | --- | --- | --- |
| **Individual randomised**  **and cross over randomised** | **Randomisation of the recruitment process** | | **Randomisation process** | | **Deviations from intended** | **Missing outcome data** | **Measurement of the outcome** | **Selection of the reported result** | **Overall Risk of bias** |
| Calderon et al (2014) |  | | LOW | | LOW | LOW | HIGH | SOME | HIGH |
| Uemura et al (2018) |  |  | SOME | | LOW | LOW | HIGH | SOME | HIGH |
| Li et al (2016) |  |  | HIGH | | LOW | LOW | SOME | SOME | HIGH |
| Gharachourlo et al (2018) |  |  | HIGH | | SOME | SOME | LOW | SOME | HIGH |
| Otilingam et al (2015) |  |  | SOME | | LOW | HIGH | LOW | SOME | HIGH |
| Mas et al (2018) |  |  | HIGH | | LOW | HIGH | LOW | SOME | HIGH |
| Fiedler et al (2019) |  |  | HIGH | | LOW | LOW | LOW | SOME | HIGH |
| Parekh et al (2017) |  |  | HIGH | | LOW | HIGH | LOW | SOME | HIGH |
| Tai et al (2016) |  |  | HIGH | | LOW | SOME | LOW | SOME | HIGH |
| Handa et al (2020) |  |  | SOME | | LOW | LOW | SOME | SOME | SOME |
| Kim et al (2020) |  |  | SOME | | LOW | LOW | LOW | SOME | SOME |
| Tavakoly Sany et al (2019) |  |  | SOME | | LOW | LOW | SOME | SOME | SOME |
| Smith et al (2019) |  |  | LOW | | LOW | SOME | SOME | SOME | SOME |
| **Cluster randomised** |  | |  | |  |  |  |  |  |
| Liu et al (2018) | HIGH | | LOW | | HIGH | LOW | HIGH | SOME | HIGH |
| Han et al (2017) | HIGH | | HIGH | | LOW | LOW | LOW | SOME | HIGH |
| Zhuang et al (2016) | SOME | | SOME | | LOW | LOW | LOW | SOME | HIGH |
| Panahi et al (2018) | SOME | | SOME | | LOW | LOW | LOW | SOME | HIGH |
| McCaffery et al (2019) | SOME | | HIGH | | LOW | LOW | LOW | SOME | HIGH |
| **Non-randomised** | **Risk of confounding** | **Selection bias** | | **Classification bias** |  |  |  |  |  |
| Mas et al (2017) | HIGH | LOW | | LOW | LOW | HIGH | LOW | SOME | HIGH |
| Tsai et al (2018) | HIGH | LOW | | LOW | LOW | HIGH | LOW | SOME | HIGH |
| Banbury et al (2020) | CRIT | LOW | | LOW | LOW | HIGH | HIGH | SOME | CRIT |
| Knudsen et al (2019) | HIGH | HIGH | | LOW | LOW | HIGH | HIGH | SOME | HIGH |

**Note:** Low = Low risk of bias, Some = Some concerns, High = High risk of bias, Crit = Critical risk of bias
